# Assays of Angiogenic Potential Using Quail and Chicken Chorioallantoic Membrane (CAM)

**DOI:** 10.1002/cpz1.70223

**Published:** 2025-11-29

**Authors:** Letícia Alves Fernandes, Gabriela Riceti Inhauser Magalhães, Ana Claudia Oliveira Carreira

**Affiliations:** ^1^ Department of Surgery, School of Veterinary Medicine and Animal Science University of São Paulo São Paulo SP Brazil; ^2^ Center for Natural and Human Sciences Federal University of ABC Santo André SP Brazil

**Keywords:** angiogenesis, chorioallantoic membrane assay, extracellular vesicles, microscopy analyses, tissue engineering, vasculogenesis

## Abstract

Angiogenesis is crucial in tissue repair, wound healing, and embryo development. Maintaining a balance between pro‐angiogenic and anti‐angiogenic factors is crucial for tissue homeostasis, thereby preventing the development of pathological conditions. In addition, the tissue microenvironment constantly influences molecular signaling, potentially altering this delicate balance. The chorioallantoic membrane (CAM) assay has emerged as a dynamic, cost‐effective, and ethically favorable alternative for studying angiogenesis. This work presents a detailed and accessible approach to the CAM assay, focusing on the evaluation of the angiogenic potential of applied stimuli (such as conditioned media, extracellular vesicles, or bioscaffolds) through various techniques, including hematoxylin and eosin, PicroSirius Red, Alcian Blue, and resorcin‐fuchsin staining; immunofluorescence; immunohistochemistry; and scanning electron microscopy (SEM). Our protocol uses the *in ovo* CAM assay approach, utilizing a simple incubator setup that eliminates the need for expensive equipment. The primary objective is to provide a comprehensive methodology for researchers to efficiently analyze angiogenesis, offering insights into vessel morphology and protein expression. The experiment comprises three major steps: (1) egg opening, stimulation, and sample collection; (2) histological processing and paraffin blocking; and (3) microscopic analyses. The first and second procedures take approximately 15 days, after which each analysis can be conducted whenever the researcher judges appropriate. The expected results include several types of data concerning the proliferation rate, vessel presence and integrity, measurement of angiogenic‐specific proteins, and investigation of structural proteins. © 2025 The Author(s). Current Protocols published by Wiley Periodicals LLC.

**Basic Protocol**: Angiogenesis quail and chicken chorioallantoic membrane assay analyses

## INTRODUCTION

Angiogenesis, the mechanisms by which new blood vessels grow from established ones, is crucial in various biological processes, such as physiological tissue repair, expansion, and remodeling in wound healing, menstruation, and embryo development (Aguiar Koga et al., 2023; Nowak‐Sliwinska et al., 2018). Angiogenesis is driven primarily by endothelial cells, which engage in cellular proliferation and migration, adapting themselves to tissue microenvironment signaling (Zhang et al., 2022). The balance between pro‐angiogenic and anti‐angiogenic molecules, known as the “angiogenic switch,” is essential for preserving tissue homeostasis and preventing pathological conditions such as cancer and inflammatory diseases (Bhat et al., 2021; Dudley & Griffioen, 2023; Zhou et al., 2022). Several techniques are available to study these mechanisms, including *in vitro*, *in vivo*, and *ex vivo* methods. *In vitro* experiments often assess endothelial cell proliferation and vessel formation through wound healing assays, tube formation assays, migration and invasion co‐culture assays, matrix degradation assays, and the development of 3D spheroids with endothelial cells and/or co‐culture. *In vivo* tests can be conducted using mice, rats, or other rodents to evaluate different areas such as the mesentery, aortic ring, corneal, fetal metatarsal, or ear angiogenesis areas (Bhat et al., 2021).

Despite the various options available, most of these experiments are expensive and technically challenging, requiring specialized personnel. Ethically, the use of animals in experimental research is becoming more difficult with efforts to minimize the number of animals to avoid unnecessary suffering (Robinson et al., 2019). To address these challenges, the chorioallantoic membrane (CAM) assay—using embryonic eggs up till the 12th day of development, which are usually thrown away in the egg industry—has emerged as a viable alternative. The first reported CAM assay dates from 1913 and demonstrated the angiogenic potential of tumor cells (Ribatti, 2004). Later, the Folkmann team helped to establish it as an easy and usable technique in 1974, and since then, numerous studies have used CAM assays to investigate angiogenesis for different applications (Ribatti, 2004; Vimalraj et al., 2019). Noteworthy applications include their use in regenerative medicine to explore the angiogenic potential of hydrogels and extracellular matrices and in cancer biology to understand cancer pathology and investigate new anti‐angiogenic molecules (Eckrich et al., 2020; Handel et al., 2013; Kohli et al., 2020; Moreno‐Jiménez et al., 2016; Palaniappan et al., 2020).

CAM assays can be performed in two ways: (i) *ex ovo* and (ii) *in ovo*. The *ex ovo* approach consists of replacing the eggshell with a petri dish or an appropriate container. In contrast, *in ovo* experiments are carried out in the eggshell. The main advantage of the *ex ovo* approach is improved membrane exposure; however, the *in ovo* approach provides higher long‐term viability and reduced chances of contamination (Ribatti, 2017, 2022). Numerous protocols reported in the literature detail the execution of both types of CAM assays to investigate angiogenesis and invasion potential under the effects of various stimuli (Borges et al., 2004; Eckrich et al., 2020; Ponce & Kleinmann, 2003; Ribatti, 2022; Ribatti et al., 2006). Notably, however, none of these articles demonstrate how to analyze results using techniques beyond traditional hematoxylin and eosin staining. With this protocol, we aim to fill this gap by providing a cheap, easy, and detailed approach to conducting CAM assays, encompassing different histological analyses, immunohistochemistry, immunofluorescence, and scanning electron microscopy (SEM). By employing this array of procedures, our primary objective is to provide robust evidence of angiogenic potential through different means. Our procedure consists of the *in ovo* approach using any incubator that maintains the temperature, with humidity provided with just a water tray, and no need for expensive materials or equipment. After the stimulation process, hematoxylin and eosin, PicroSirius Red, Alcian Blue, and resorcin‐fuchsin staining are carried out using kits or in‐house protocols. Also, we have established an easy procedure to analyze specific proteins through immunofluorescence and immunohistochemistry. Finally, we demonstrate an efficient method to evaluate vessel morphology using SEM technology.

Even though this protocol can be performed by anyone, a few limitations must be considered. The major limitation is the need to acquire quail and especially chicken eggs with a good fertility rate. A partnership with professional producers that can provide a high number of eggs with good embryonic control is recommended to mitigate this problem. Although the primary aim is to provide different analyses of the CAM assay, another possible challenge is the availability of scanning and fluorescence microscopes, if needed.

This protocol is recommended as a means to investigate the formation of blood vessels in multiple biological samples. Any type of material can be applied to the membrane, on top of filter paper or directly. Based on our experience in our laboratory, this CAM assay method is a useful option to analyze the angiogenic potential of conditioned medium, extracellular vesicles, hydrogels, and native and decellularized extracellular matrix from different tissues.

*CAUTION*: To avoid contamination, the incubator and hood must be cleaned using 70% ethanol. All materials used in opening the eggs should be properly sterilized before starting the procedure, except for the specific materials that are not autoclavable. For those kinds of materials, proper cleaning using 70% ethanol and UV light exposure should be performed.

*CAUTION*: Once you have the eggs, try to incubate them immediately; storage is not recommended, especially on warm days. If necessary, store eggs at room temperature (20°C‐22°C) and away from sunlight for up to 15 days.

*CAUTION*: Our goal is to provide a global protocol; however, adjustments may be needed according to each laboratory's conditions and equipment. To do that, the authors strongly suggest that researchers run a pilot experiment to validate the complete procedure, from the opening step to the microscopy analyses, before using precious or rare samples.

## STRATEGIC PLANNING

The first step toward effective experimental design is to consider the following questions: (1) What type of stimuli will be applied to the membrane? (2) Could different stimuli be tested? (3) What type of control should be used for each stimulus? (4) How much time will the stimulus be tested in the membrane? Once all of these questions are answered, define the number of eggs needed for the experiment and plan experimental days so as to avoid egg loss.

*NOTE*: All protocols involving animals must be reviewed and approved by the appropriate Animal Care and Use Committee and must follow regulations for the care and use of laboratory animals.

## QUAIL AND CHICKEN CHORIOALLANTOIC MEMBRANE ASSAY OF ANGIOGENESIS

**Figure 1:**
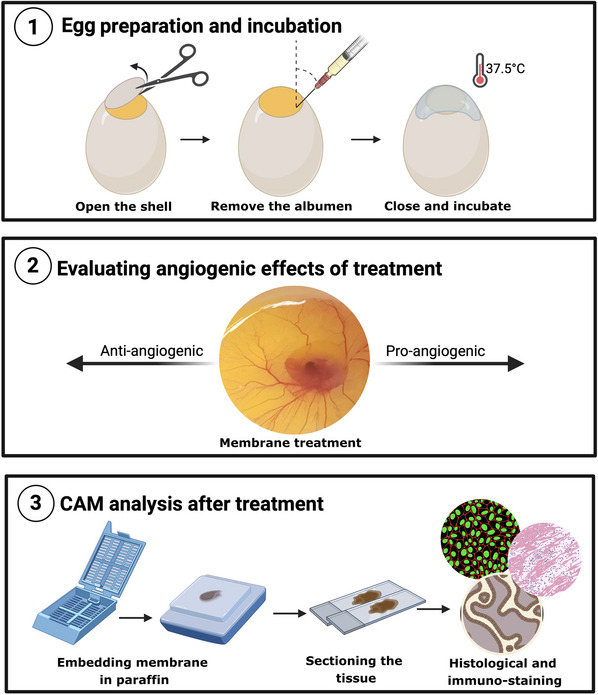
Illustrative workflow demonstrating the procedures described in this protocol, starting from (1) egg opening and incubation procedure, (2) treatment approach, and (3) histology analyses to evaluate angiogenesis potential.

This protocol presents a cost‐effective and detailed approach to conducting the CAM assay, incorporating histological analyses, immunohistochemistry, immunofluorescence, and scanning electron microscopy (SEM). We will describe how to perform the experiment using the *in ovo* approach. After stimulation, different staining techniques, immunohistochemistry, and immunofluorescence are described for protein analysis (Fig. 1). Finally, we detail an efficient SEM‐based method for evaluating vessel morphology, offering robust evidence of angiogenic potential.

### Materials

70%, 80%, and 90% (v/v) ethanol (see recipe)
Distilled water, sterile
Phosphate‐buffered saline (PBS; see recipe)
Stimuli to be tested (e.g., conditioned media, extracellular vesicles, bioscaffolds)
4% paraformaldehyde (PFA; see recipe)
Paraffin (EP‐21‐20066, EasyPath, Brazil)
100% ethanol: ethanol P.A. 99.9% (cat. no. 13‐1084‐50, LGC Biotecnologia, Brazil)
Xylene (LabSynth, cat. no. 256407)
Hematoxylin (cat. no. HH08362SO, Êxodo Científica, Brazil)
Eosin (cat. no. P0101020000381, Dinâmica, Brazil)
Alcian Blue HistoKit (cat. no. EP‐11‐20018, EasyPath, Brazil)
Picro Sirius Red HistoKit (cat. no. EP‐11‐20011 EasyPath, Brazil)
Resorcin‐fuchsin (see recipe)
Toluene solution (cat. no. UN1294, Fisher Chemical)
Citrate‐phosphate buffer (see recipe)
EnVision Flex Kit (Dako, Agilent Technologies, California, EUA)
BSA wash solution (see recipe)
2% and 0.5% (v/v) bovine serum albumin (BSA; see recipe)
3,3′‐Diaminobenzidine (DAB) visualization solution (see recipe)
Primary antibodies (depending on your research question): e.g.,
  Anti‐MMP2, diluted 1:100 (Abcam, cat. no. Ab37150)
  Anti‐collagen I, diluted 1:200 (ThermoFisher, cat. no. PA5‐29569)
  Anti‐vimentin, diluted 1:100 (Abcam, cat. no. Ab92547)
Secondary antibodies (depending on your research question): e.g., Alexa Fluor 488–anti‐rabbit antibody, diluted 1:200 (ThermoFisher, cat. no. A114008)
Permeabilization solution (see recipe)
4′,6‐Diamidino‐2‐phenylindole (DAPI; cat. no. D3571, ThermoFisher Scientific)
Anti‐fading mounting medium (e.g., ProLong antifading mounting medium, cat. no. P36934, ThermoFisher Scientific, or Vectashield antifade mounting medium, cat. no. H‐1000‐10, Vector Laboratories)
Immunofluorescence (IF) buffer (see recipe)
Nail polish (any color or brand)

Egg incubator or any common incubator with temperature settings available
Hygrometer and thermometer
36‐position vial racks (cat. no. 868805, Wheaton, DWK Life Sciences)
Laminar‐flow hood
Beaker of appropriate size for the number of samples
Iris scissors (any brand)
5‐ or 10‐ml syringe (any brand)
0.8 × 40‐mm or 1.2 × 40‐mm needles
Fine‐point straight dissecting forceps (any brand)
Cotton swabs
Refrigerator, 4°C
Micro spatula (cat. no. HS15906, Merck), for weighing
Weighing flask
10‐µm‐thick plastic wrap (any brand), cut into 3 × 3‐cm pieces for quail eggs or 6 × 6‐cm pieces for chicken eggs
35‐mm petri dishes (any brand)
0.83‐mm‐thick filter paper (any brand), cut into 0.5 × 0.5‐cm pieces for quail eggs or 1 × 1‐cm pieces for chicken eggs
20‐µl automatic pipet (Pipetman L, Gilson, USA)
Stereo zoom microscope (e.g., M80, Leica Microsystems, Germany)
28 × 40 × 6.8‐mm histology cassette with 5 × 1‐mm holes
Shaker (no temperature control necessary)
Histology paraffin mold
Microtome apparatus (RM2125RT, Leica Microsystems, Germany)
Paraffin incubator (002CB, Fanem, Brazil), set at 60°C
26 × 76‐mm histology slides (Exacta, Brazil; for routine histological analyses) and/or silanized slides (Starfrost, cat. no. 112711, Knittel Glass, Germany, or Silane‐Prep Slides, Millipore Sigma; for immunochemical analyses)
Dark box for slides (cat. no. EP‐51‐05022, EasyPath, Brazil)
24 × 50‐mm histology coverslips (Exacta, Brazil)
Multi‐well dish
Critical‐point dryer (EM CPD300, Leica Microsystems, Germany)
Scanning electron microscope (Leo 435VP, Zeiss, USA)
Carbon tape appropriate for SEM analysis
Microwave oven with current 13.5 A and frequency 2450 Hz (MEF41, Electrolux, Brazil)
Hydrophobic pen (EasyPath, Brazil)
Fluorescence microscopy (DM6 B Upright, Leica Microsystems, Germany)

#### Egg Opening and Stimulation

##### Cleaning and setup of the incubator

1 Clean the incubator with 70% ethanol.
2 Place a water tray containing 1‐2 L of sterile, distilled water inside the incubator.
  We use an incubator without an automatic humidity control system. If you have one that adjusts humidity automatically, set it to 50% and omit step 2.
3 Set the incubator temperature to 37.5°C.
  We recommend the use of an additional thermometer and hygrometer to guarantee proper temperature and humidity settings.

###### Cleaning and incubation of eggs

**Figure 2:**
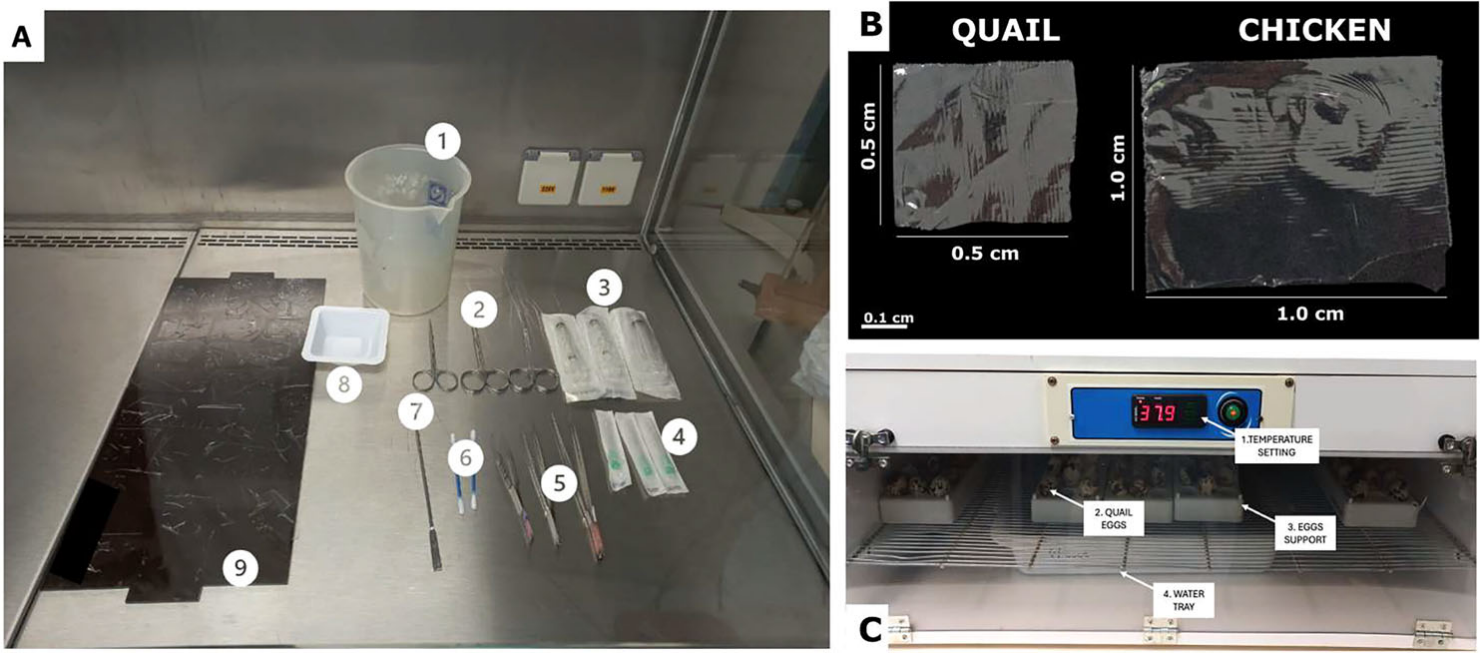
(**A**) Examples of essential materials used in the opening step (1, waste beaker; 2, scissors; 3, syringe; 4, needles; 5, forceps; 6, cotton swab; 7, spatula; 8, weighing flask; 9, plastic wrap laid out to form a flat pad). (**B**) Example of how to cut the plastic film to cover eggs according to species (quail, 0.5 cm × 0.5 cm; chicken, 1 cm × 1 cm). (**C**) Example of the egg incubator used in our laboratory, demonstrating the temperature setting, quail eggs, egg support racks, and water tray used to maintain relative humidity.

**Figure 3:**
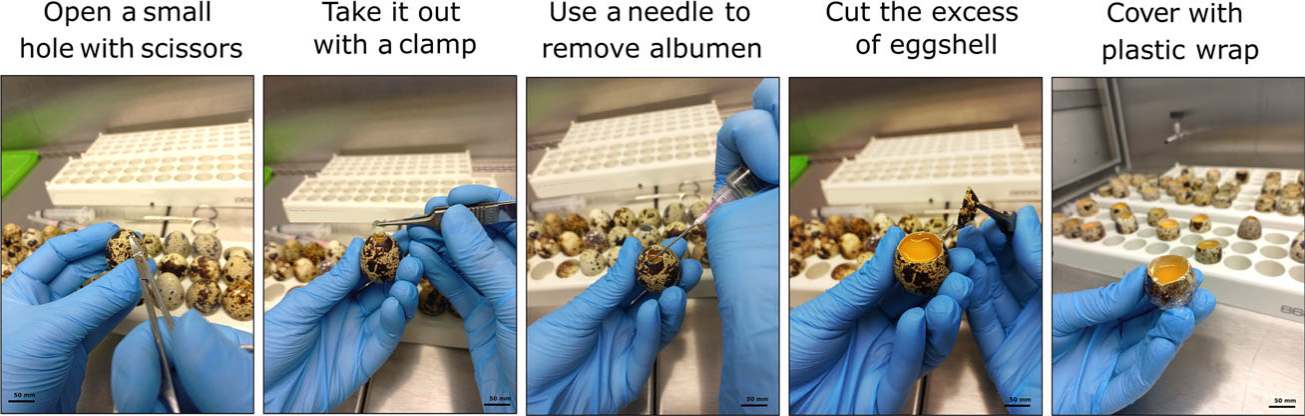
Demonstration of the steps of the opening procedure using quail eggs.

4 Clean each egg with a soft paper or cloth moistened with 70% ethanol.
  During the cleaning process, ensure the paper/cloth is not saturated with 70% ethanol, as this may cause embryo intoxication.
5 Place the eggs on a clean rack (e.g., 36‐position Wheaton vial rack) to ensure proper handling and avoid contamination.
  The rack is used to hold eggs during incubation; examples are shown in Figure 2C and Figure 3.

6 Incubate the eggs in the egg incubator until day 3.

###### Egg opening

Figure 2 illustrates how to prepare the laminar‐flow hood and incubator.

**Figure 4:**
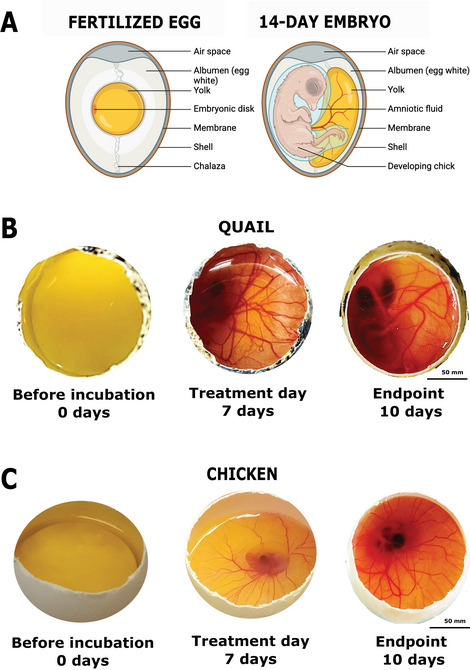
(**A**) Illustration demonstrating essential tissues during the embryo development process. Representative images from (**B**) quail and (**C**) chicken embryo development at days 0, 7, and 10.

7 On day 3, prepare the laminar flow and all necessary materials (scissors, syringe, needles, forceps, cotton swab, spatula, weighing flask, and plastic wrap; Fig. 2), disinfecting all materials with 70% ethanol before introducing them into the hood to minimize the risk of contamination. Apply UV light for at least 5 min before starting the opening process.
8 Once all materials and the working area have been properly prepared, open up the eggs one by one. For each egg, use forceps to create a small access point in the shell, allowing you to insert the scissors. Cut carefully until an adequate observation window is obtained.
  Steps 8‐11 are illustrated in Fig. 3.
  As chicken eggs may be slightly difficult to open, gently tapping the shell with the opposite end of the forceps can assist in the process.
9 Using the syringe with a needle, remove enough of the albumen (clear liquid inside the egg) to expose an adequate surface area for embryo development. The volume needed depends on the egg size: ∼2‐3 ml for quail eggs and ∼5‐6 ml for chicken eggs. Then, cut the eggshell off at the level of the remaining contents using scissors. Be sure to leave ∼0.5 cm of shell as a protective margin to prevent leakage.
10 Using a cotton swab soaked in sterile PBS, gently moisten the entire eggshell.
11 Immediately cover the top opening with a piece of plastic wrap and seal securely (the PBS will help the plastic adhere to the eggshell).
  The plastic wrap should be prepared as demonstrated in Figure 2B, depending on the size of the eggs, and then cleaned with 70% ethanol and exposed to UV light for 5 min. We recommend cutting up and cleaning all necessary plastic wrap before starting the procedure (as described in step 7).
12 After you have opened up all of the eggs, place them back in the incubator and incubate them until day 7.
13 Embryos should be evaluated daily from day 3 to day 7. Healthy embryos will exhibit a heartbeat, a prominent, bright red vascular network, and consistent growth over the days, as illustrated in Figure 4. Discard any eggs that do not develop.
  The rate of embryo loss may vary depending on the origin and quality of the eggs. Generally, it is ∼20% for quail eggs and can reach up to 40% for chicken eggs. Therefore, it is recommended that the experiment be designed with at least 5‐6 biological replicates per condition to ensure adequate statistical power at the end of the experiment.

###### Treatment and collection of the chorioallantoic membrane

14 Divide the filter paper squares (0.5 cm × 0.5 cm for quail eggs or 1 cm × 1 cm for chicken eggs) into petri dishes according to the treatment(s) (stimuli) to be applied.
  These filter papers will be used to apply the treatment onto the embryonic membrane.
  Each quail egg membrane can hold up to three treatment‐loaded filter papers and each chicken egg up to four. Ideally, all treatments in one egg should be from the same experimental group to avoid cross‐interference. In chicken eggs, however, the larger surface area allows sufficient spatial separation between application sites to allow the use of different treatments if necessary (e.g., when the number of viable eggs is small).
15 Use a pipet to dispense the appropriate treatment solution onto each filter paper square and allow it to penetrate for 5‐10 min before proceeding. The smaller filter paper squares used for quail eggs have a maximum capacity of 10 µl of solution each, whereas the larger ones used for chicken eggs can hold up to 20 µl of solution.
  Filter paper is essential for the delivery of liquid stimuli. However, alternative materials such as tissue fragments can be placed directly onto the membrane, as long as they fit the same specifications in regard to size (see step 14) and loading capacity, and are adequately sterilized.
16 Once the treatments are prepared, transfer the eggs for one experimental group into the laminar flow hood.
  We suggest separating the eggs into groups in this fashion so that you can work with a few at a time, because the eggs should not be kept out of the incubator for too long.
17 Remove the plastic wrap.
18 Using straight tissue forceps, transfer each filter paper square (stimulus) to the membrane of the selected egg, placing it on top of a blood vessel.
  Use similar blood vessel structures for both the control and treatment.
19 As described in steps 10 and 11, moisten a cotton swab with sterile PBS buffer and carefully apply it around the egg before resealing with a fresh piece of plastic wrap.
20 Return the eggs to the incubator, maintaining the same temperature and humidity conditions as initially set.
21 Repeat steps 16‐20 for the other experimental groups.
  Eggs can be evaluated and membranes collected from day 8 to day 12, depending on your experimental design and research aims.
  Figure 4 illustrates the developmental progression of quail and chicken eggs throughout the experimental period (see Video 1 and Video 2 to view chicken embryos filmed at day 3 and day 10, respectively).
  For quail eggs, after day 11, the embryo development accelerates, and the membrane begins to disappear. For chicken eggs, evaluations can be performed up to day 13.
22 *Egg euthanasia*: To stop embryo development, using a syringe and needle, apply ∼2 ml of 4% PFA inside the egg next to the embryo. Then, spread 4% PFA on top of the membrane to facilitate collection of the membrane. Place the egg in the refrigerator (4°C) and leave overnight.

**Video 1:** Chicken embryo at day 3.

**Video 2:** Chicken embryo at day 10.

**Figure 5:**
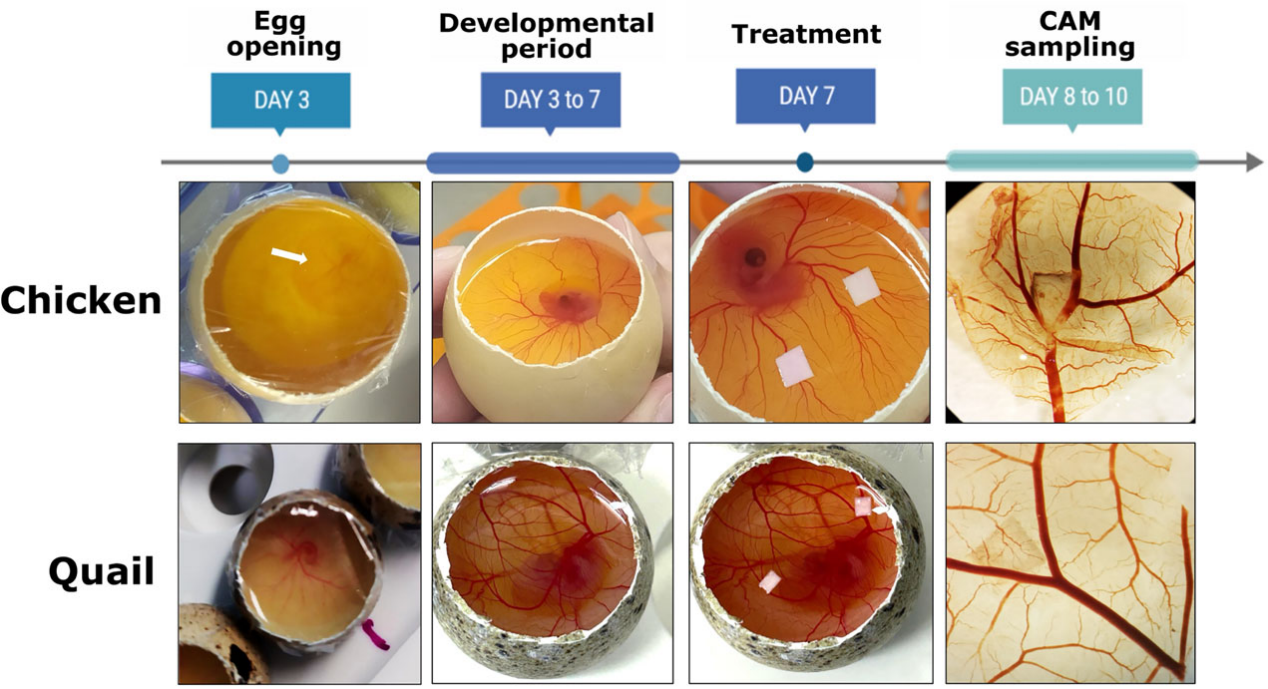
Timeline of embryo development with demonstrative images at each time experiment time point: day 0 (egg opening); days 3‐7, during which only observation and maintenance are required; day 7, the treatment day, when stimuli are applied to the membrane; and the final day, when the membrane is collected that can occur on days 8‐10.

23 *Membrane collection*: Collect the membrane around the filter paper (treatment) using scissors. Store the membranes in a multi‐well dish or a single 35‐mm petri dish, covered with 4% PFA, for another 24 hr.
*IMPORTANT NOTE*: Steps 22 and 23 are critical and must be performed with care.
  After collection, membranes may be imaged macroscopically using a stereomicroscope.
24 Depending on your analysis framework, immerse the membranes in sterile PBS (for immunohistochemistry, immunofluorescence, and histological staining) or 70% ethanol (for SEM).
  Membranes for each stimulus group need to be collected individually. During collection, special attention must be paid to avoid mechanical damage to the membrane.
  For SEM analysis, it is crucial to avoid damaging the membrane. Use forceps as carefully as possible and position them away from the region of interest (treatment area), as SEM imaging will reveal even the slightest structural defects.
  Figure 5 illustrates the experimental timeline.
  The procedure may be paused here and the histologic processing and analyses done at a later time. However, keeping membranes in storage for much longer than 1 month is not recommended.

###### Preparation of chorioallantoic membrane for histological processing

**Figure 6:**
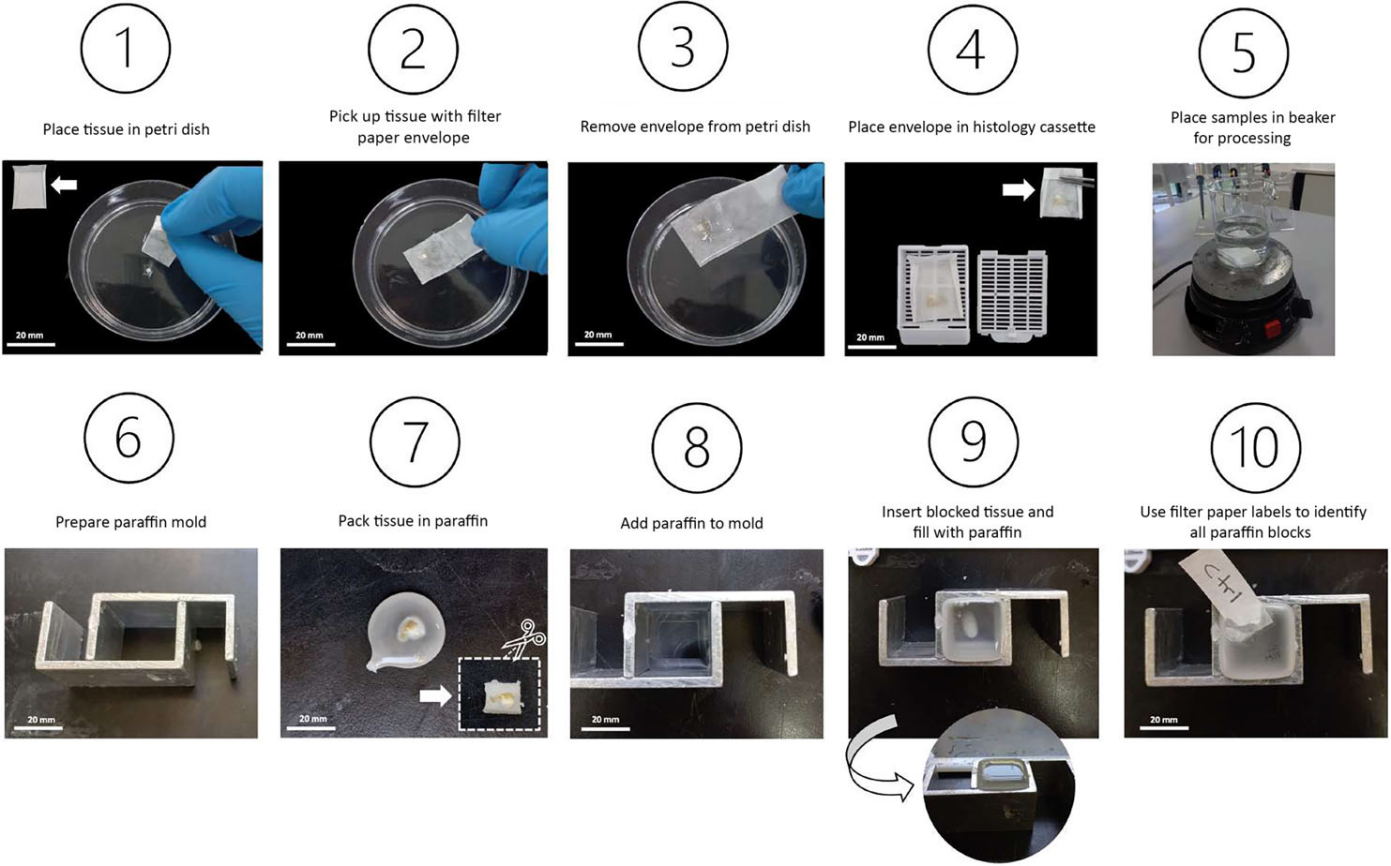
Examples of the steps of CAM histological processing.

Figure 6 provides a step‐by‐step illustration of the histology procedures.

25 For histological analysis, the membranes must remain as flat as possible. To do this, cut a square of filter paper large enough to fully accommodate the membrane, leaving extra space around the edges to fold the paper and form an envelope. Set the paper aside for later use.
26 Gently remove the membranes from PBS using a micro spatula. Place the membrane into a petri dish containing 70% ethanol.
  This step helps to keep the membrane flat before it is inserted into the filter paper envelope.
27 Using the pre‐cut filter paper envelope, gently fish the membrane out of the ethanol bath. Position the membrane at the center of the paper and carefully fold in all sides to form a closed envelope. Ensure that the membrane remains flat and properly positioned throughout the process (Fig. 6 illustrates this step in detail).
  Do not fold or wrinkle the membrane when closing the filter paper. Handle it gently to maintain its integrity.
28 Place each filter paper envelope (containing the membrane) into a histology cassette to maintain the membranes intact during the process. Place each cassette in a beaker to perform the next steps.

###### Histological processing

Membrane dehydration and clearing are performed through successive ethanol and xylene washes, all conducted within the beaker prepared in step 28. These washes must be done on a shaker to maintain liquid homogeneity. To begin this procedure, prepare two beakers with paraffin and place them in an incubator set at 60°C to melt. Proceed only once the paraffin is fully melted and ready.

29 Dispense 70% ethanol into the beaker until all histology cassettes are fully submerged. Place the beaker on a shaker for 10 min to ensure uniform washing.
30 After the incubation, remove 70% ethanol and repeat the procedure using 80% ethanol for 10 min, followed by 90% and 100% ethanol for 10 min each. Perform a second wash with a fresh 100% ethanol solution for an additional 10 min.
31 Remove all ethanol from the beaker, replace it with xylene, and let stand 5 min to wash.
32 Perform a second wash with fresh xylene solution for an additional 10 min.
33 Using forceps, transfer all cassettes into a fresh beaker filled with paraffin and incubate for 1 hr. Then, transfer them to the second beaker containing fresh paraffin and incubate for an additional hour.
  The first wash typically contains more impurities and is less effective; therefore, a second wash must always be performed using fresh solution. After use, all reagents can be filtered using filter paper and stored for future reuse up to five times, as long the solutions remain clear.
  After removing the tissue from the second paraffin container in step 33, proceed to embed the tissues. Each cassette should be opened individually, and the tissue carefully removed from the filter paper envelope. Follow the steps below and refer to Figure 6 for guidance.
34 Using an appropriate surface (typically provided by the histology laboratory; if unavailable, a wooden platform is sufficient), create a small paraffin circle where the membrane will be placed as flat as possible, and allow it to dry.
  Keep the membrane as flat as possible.
35 Prepare the paraffin mold with sufficient space to accommodate the membrane in a vertical position. Fill the mold halfway with paraffin, insert the small paraffin circle prepared in step 34 vertically into the mold, and immediately fill the remainder of the mold with paraffin.
*IMPORTANT NOTE*: This process is critical and should be performed quickly to avoid solidification of the paraffin and loss of the material. Because of the high temperature, using forceps is helpful.
36 Allow the paraffin block to solidify at room temperature for at least 12 hr, keeping the block within the embedding mold.
37 Section the paraffin blocks in a microtome at 5‐µm thickness and transfer sections to slides.
  For routine histological staining, tissue sections can be placed on regular glass slides. However, for immunochemical staining techniques, such as immunohistochemistry (IHC) and immunofluorescence (IF), it is important to use coated slides (e.g., Silane‐Prep Slides, Millipore Sigma) to ensure that tissue sections remain adhered during the staining process.
38 Place slides in an incubator at 60°C for ∼30 min before storing.
39 Store both sets of slides (normal slides for histological staining and coated slides for immunostaining) until the respective analysis procedures are performed.
  Sectioning can take several days, depending on the number of samples and slides involved.
  It is important to section the paraffin until the tissue is visible within the block. If you are uncertain about whether tissue is present, you can place one slide in the incubator at 60°C for ∼20 min and then check it under a light to confirm the presence of the tissue.
  The procedure can be paused here without any concern and tissue sections stained at a later time. After paraffin embedding, tissue can be stored, with proper identification.

#### Histological Staining

##### Slide preparation for histological staining

40 Before starting the staining process, retrieve the slides from storage and place them in an incubator at 60°C for up to 2 hr to melt, removing the excess paraffin. If processing fresh samples, slides should also be incubated under the same conditions.
41 Place the slides in a slide rack for histology and begin the staining process by immersing the slides in xylene for 5 min.
42 Transfer the slide rack to fresh xylene for 5 min.
43 Next, immerse the slide rack in 100% ethanol for 3 min, and repeat.
44 Immerse the slide rack successively in 95% ethanol and 70% ethanol for 3 min each.
  From this point on, follow the steps appropriate for the type of staining you are performing.
45a For hematoxylin and eosin staining:
  i. Place the slide rack in water for 10 min.
  ii. Place the slide rack in hematoxylin for 2 min.
  iii. Wash with water for 5 min.
  iv. Place the slide rack into the eosin for 15‐30 s, depending on the integrity and age of the eosin (older solutions may require longer staining times).
45b For Alcian Blue staining:
  i. Place the slide rack in water for 1 min.
  ii. Place the slide rack in water for 1 min.
  iii. Place the slide rack into Alcian Blue, pH 2.5 (Reagent A from the Alcian Blue HistoKit) for 30 min.
    We have also tested homemade Alcian Blue, which performed as well as the commercial kit used.
  iv. Place the slide rack in water for 1 min.
  v. Place the slide rack in hematoxylin (Reagent B from the Alcian Blue HistoKit) for 5 min.
  vi. Place the slide rack in water for 1 min.
45c For PicroSirius Red staining:
  i. Place the slide rack in water for 3 min.
  ii. Place the slide rack in PicroSirius Red (Reagent A from the PicroSirius HistoKit for 1 hr), followed by a water bath for 3 min.
  iii. Place the slide rack in hematoxylin (Reagent B from the PicroSirius HistoKit) for 4 min.
45d For resorcin‐fuchsin staining:
  i. Place the slide rack in water for 5 min.
  ii. Immerse the slide rack in resorcin‐fuchsin for 1 hr, followed by a water bath for 5 min.
  iii. Wash the slides by placing the slide rack in 70% ethanol for 5 min. Repeat twice more using fresh 70% ethanol each time.
    From this point on, the final steps of histological staining (mounting procedure) are the same for all staining procedures.

###### Mounting of stained histological slides

46 Using the slide rack, perform two quick immersions (“baths”) in 95% ethanol, followed by two baths in 100% ethanol.
  The term “bath” here refers to quickly immersing the rack in the solution and removing it.
47 After the baths, place the rack in xylene for 5 min, and repeat this step using a fresh xylene solution.
  After these steps, samples should remain in xylene to avoid degradation. However, the next steps need to be done as quickly as possible.
48 First, carefully dry the slides with paper to make them as clean and dry as possible.
49 Using a Pasteur pipet, apply five drops of toluene solution (histology glue) at random points of the slide. The goal is to spread the glue evenly across the slide, ensuring sufficient coverage to prevent air bubbles.
50 Carefully place the coverslips on top, taking care to avoid trapping air bubbles. Do not press directly on the tissue at any time, as they are fragile and may tear.
51 Allow slides to dry at room temperature for 3‐5 days.
  Because the tissue sections are very small and thin, images will probably be obtained using 20× or 40× microscopy objectives. Therefore, slides should be thoroughly dried to avoid tissue tearing during the image capture process.
  We use Alcian Blue staining (HistoKit EasyPath Diagnostics, Brazil) and PicroSirius Red staining (HistoKit EasyPath Diagnostics, Brazil). However, the procedure can be adapted according to each laboratory's technique, using the same xylene and ethanol incubation times described here, which are the most critical steps. A homemade Alcian Blue solution was also tested as a substitute for the kit, using the same incubation time, and performed equally well.

###### Imaging analysis

Any or all of the following procedures may be performed, depending on the experimental design.

###### Scanning electron microscopy

As previously described (step 24b), samples for SEM will be stored in 70% ethanol until analysis.

52 Use a Pasteur pipet to remove the 70% ethanol from samples in the multi‐well dish (the number of wells will depend on the number of samples)
53 Add 1 ml/well of 80% ethanol to the multi‐well dish (for a 24‐well dish) and let stand for 2 min. (Use 3 ml per well if using a 6‐well dish or 0.5 ml per well if using a 48‐well dish.)
54 Use a Pasteur pipet to remove the 80% ethanol.
55 Add 1 ml of 90% ethanol for 2 min
56 Remove the 90% ethanol with a Pasteur pipet.
57 Add 1 ml of 100% ethanol for 2 min.
58 Remove the 100% ethanol.
59 Add 1 ml of 100% ethanol for an additional 2 min.
  All washes need to be performed inside the multi‐well dish, as described in step 53.
60 Place samples in a filter paper envelope as described for histology processing (steps 25‐27) and dry them in a critical‐point dryer using the following parameters: speed in medium mode for CO_2_ input, 12 cycles of CO_2_ exchange for 3 seconds, heat at 40°C for CO_2_ exit at medium mode.
  For SEM analysis, the tissue must be completely dried to ensure proper vacuum formation within the equipment. A critical‐point dryer is used to dehydrate the samples while preserving their surface integrity, which is essential for obtaining high‐quality SEM images.
61 Once the supercritical point is reached, remove samples carefully from the envelope using forceps as described for histology processing (step 27 annotation).
62 Using carbon tape appropriate for SEM analysis, cut a small piece and place it on an SEM stub
63 Remove the protecting tape and place the membrane onto the SEM stub.
  The next steps include gold coating (for example, using an automatic scatter coater and sputtering system, e.g., Emitech K550, Quorum Technology) and SEM imaging; however, both procedures may be performed by a specialized technician, as SEM equipment is rarely operated by general users.

###### Immunohistochemistry (IHC) assay

64 Before the staining process is begun, the stored slides should be placed in a slide rack and incubated at 60°C for up to 2 hr to melt the excess paraffin.
65 In sequence, perform xylene and ethanol washes as described in steps 41‐44 to prepare slides for IHC.
66 Maintain the slide rack in a water bath for 5 min to remove excess ethanol.
67 Antigen retrieval: Place the slides horizontally in a plastic container. Add enough citrate‐phosphate buffer to fully cover all slides and tissue sections, then microwave for 35 s.
  A disposable pipet tip box serves as an ideal plastic container for this procedure.
  We use a microwave with a current of 13.5 A and a frequency of 2450 Hz.
68 Immediately wash slides with cold (4°C) PBS buffer (pH 7.2‐7.4) twice, for 5 min each time, to cool the sample.
69 Use paper or cotton to dry slides, and circle each tissue section using a hydrophobic pen.
70 Apply a 10‐µl drop of blocking reagent on top of each section and incubate at room temperature for 10 min (to block endogenous peroxidase).
  We like to use the EnVision Flex Peroxidase Blocking Reagent (Dako), but this procedure can be performed using different reagents that do not involve commercial kits, if necessary.
71 Apply a drop of BSA wash solution using a 20‐ or 200‐µl pipet. Incubate 5 min and then repeat the process once more.
72 Drip 10 µl of 2% BSA onto each section and incubate at room temperature for 30 min (to block nonspecific sites).
73 Wash twice with 0.5% BSA for 5 min each time
74 Incubate the slides with primary antibody overnight using the dilution indicated in the datasheet (∼16 hr).
  Primary antibodies should be diluted in 2% BSA.
75 Wash twice with 0.5% BSA for 5 min each time.
76 Incubate slides with 10 µl of EnVision FLEX/HRP Reagent (HRP‐conjugated secondary antibody; from EnVision kit) for 30 min.
77 Wash twice with 0.5% BSA for 5 min each time.
78 Place 10 µl of DAB visualization solution on each section and let stand 5 min (maximum).
  To visualize DAB's action, use a stereomicroscope and process one tissue section at a time. The DAB product will turn the tissue brown. If you do not see any color change after 5 min, it indicates that the specific antibody has not resulted in staining.
79 Stop the DAB reaction by using a Pasteur pipet (or a 20‐ or 200‐µl pipet) to wash the tissue twice with a drop of distilled water. While performing the DAB reaction on other tissue sections, keep the completed slides immersed in water on a slide rack.
80 After finishing all tissue sections, place the slides back into the slide rack and briefly immerse them in water to remove excess DAB (for the water bath, use any container that properly fits the slide rack and allows full immersion of all tissue sections).
81 Place slides in hematoxylin for 3 min (counterstaining).
  If working with new hematoxylin, you may need to adjust the time to be shorter to avoid excess staining that can compromise your antibody results.
82 Proceed with mounting slide washes as described in steps 47‐52 (for mounting of histology slides).
83 Allow the slides to air‐dry for ~2 days and then image slides.

###### Immunofluorescence assay

84 The initial steps for immunofluorescence are identical to those for IHC, including slide preparation and antigen retrieval; therefore, follow steps 64‐69 as previously described.
85 Drip 10 µl permeabilization solution onto each section and let stand 10 min.
86 Wash with PBS (room temperature) twice for 5 min each time using an automatic or Pasteur pipet.
87 Drip 10 µl of 2% BSA onto each section and let stand 1 hr (to block nonspecific sites).
88 Prepare primary antibody solution at an appropriate dilution and incubate slides with the solution according to the manufacturer's datasheet (this will be the same procedure as that for IHC in step 74).
  We recommend incubating slides inside a dark box at 4°C when the manufacturers call for overnight incubation.
89 Wash twice with PBS at room temperature for 5 min each time.
90 Incubate slides with 10 µl secondary antibody for 1 hr.
  Secondary antibodies should be diluted in 2% BSA, as specified in the manufacturer's datasheet.
  The antibody volume should be adjusted according to tissue section size.
91 Wash twice with IF buffer (room temperature) twice for 5 min each time.
92 Drip 10 µl of DAPI solution for 10 min.
93 Wash with PBS (room temperature) twice for 5 min each time.
94 Mount slides using an anti‐fading mounting medium.
  We usually use ProLong antifading mounting medium (cat. no. P36934, ThermoFisher Scientific) or Vectashield antifade mounting medium (cat. no. H‐1000‐10, Vector Laboratories).
95 Seal the edges of the slides using nail polish and allow the slides to air‐dry for ~2 days before imaging.
96 Image slides.

## REAGENTS AND SOLUTIONS

### BSA, 2% and 0.5% (w/v)

1 g bovine serum albumin (BSA; Sigma Aldrich, cat. no. A4737)
50 ml phosphate‐buffered saline (PBS; see recipe)
Store up to 3 months at 4°C
Filter using a 0.22‐µm‐pore‐size filter before use

To make a 0.5% BSA solution, either prepare as above but using 0.25 g BSA, or dilute 2% BSA stock solution 1/4 in PBS (either use previously filtered PBS or filter the 0.5% BSA solution after dilution).

BSA solutions are used in immunostaining protocols to prevent nonspecific protein binding. This solution is used in various stages of this protocol, with varying BSA concentration (2% and 0.5%). Because of its high protein content, there is a risk of contamination, so it is essential to prepare the solution using sterile PBS and store it at 4°C in a sterile flask.

### Citrate‐phosphate buffer solution

First, prepare the following two solutions:

Solution A: Mix 4.2 g anhydrous citric acid and 200 ml distilled water.
Solution B: Mix 52.92 g tribasic sodium citrate and 1.8 liters distilled water.
Measure the pH of solution B and adjust it by adding drops of solution A until the pH is 6.0 (this usually requires ~10% of the total solution A). Store at 4°C.
Citrate‐phosphate buffer can be prepared in advance and stored up to 1 week (prepare a small volume, if necessary). However, once the solution has been used, it should be discarded. To minimize errors, it's advisable to prepare the buffer in a sterile flask with clean water, although creating a completely sterile solution is not mandatory.

This buffer is used for antigen recovery in immunohistochemistry and immunofluorescence. A ready‐to‐use phosphate‐citrate buffer is available on the market, and it was also validated in this protocol (P4809‐50TAB, Sigma Aldrich).

### DAB visualization solution

1 drop of DAB solution (EnVision FLEX Systems, Agilent, cat. no. GV82311‐2GV)
1 ml of DAB substrate (EnVision FLEX Systems, Agilent, cat. no. GV82311‐2GV)
  This recipe is based on the manufacturer's recommendation.

### Ethanol solutions

700 ml ethanol P.A. 99.9% (v/v) (LGC Biotecnologia, cat. no. 13‐1084‐50)
300 ml distilled water
Use an alcoholometer to adjust to the proper dilution
Store in a hermetically closed flask at room temperature
  Ethanol solutions are used to perform dehydration and hydration of tissue sections before histology staining. It should be prepared at each of the concentrations called for in the protocol (70%, 80%, and 90%). Follow the example given above for 70% (v/v) ethanol.

### Fuchsin‐resorcin solution

Mix:

2 g basic fuchsin (Êxodo Científica, cat. no. FB0929RA)
4 g resorcin (Dinâmica, cat. no. P.100894)
200 ml distilled water
Heat the solution to boiling.
Add 50 ml of 30% ferric chloride (Êxodo Científica, cat. no. 39676)
Boil for 4 min.
Allow the solution to cool to approximately room temperature and filter using filter paper.
Discard the filtrate and allow the deposit retained on the filter paper to dry.
Heat 200 ml of 95% (v/v) ethanol and use this to dissolve the deposit on the filter paper.
Allow the resulting solution to cool.
Add 2 ml hydrochloric acid (HCl).
Store up to 1 year at 4°C.
  The fuchsin‐resorcin solution is used for histological staining to enable the visualization of elastic fibers. Preparation of this solution does not require sterile conditions.

### Immunofluorescence (IF) buffer

200 µl Triton X‐100 (0.2% [v/v], LGC Biotecnologia, cat. no. 13‐13315‐05)
50 µl Tween 20 (0.05% [v/v], Êxodo Científica, cat. no. 2271)
99.75 ml PBS (see recipe)
Store up to 3 months at 4°C
Filter using a 0.22‐µm‐pore‐size filter before use

### Karnovsky solution

50 ml of 4% PFA (see recipe)
50 ml of 25% glutaraldehyde solution (Merck, cat. no. ZC814039)
Filter using a 0.22‐µm‐pore‐size filter before using it.
  This solution can be prepared at any volume, maintaining a 1:1 (v/v) ratio of the components.

### Paraformaldehyde (PFA), 4%

Working in a fume hood, combine the following in a glass beaker:

40 g paraformaldehyde (PFA) P.A. (Dinâmica, cat. no. P.10.0804)
1 L PBS (see recipe)
Stir at 60°C until completely dissolved.
With continued stirring, use 1 M NaOH to adjust the pH to 7.2‐7.4.
Store in a plastic flask.
Filter using a 0.22‐µm‐pore‐size filter before use.
  Paraformaldehyde is a fixation solution used for euthanasia of chicken and quail embryo and to conserve the membrane vessels.

### Permeabilization solution

500 µl Triton X‐100 (0.5%; LGC Biotecnologia, cat. no. 13‐13315‐05)
99.5 ml IF buffer (see recipe)
Store at 4°C for up to 3 months.
Filter using a 0.22‐µm‐pore‐size filter before use.

### Phosphate‐buffered saline (PBS)

8 g NaCl (137 mM, Nova Biotecnologia, cat. no. 13‐1060‐05)
0.2 g KCl (2.7 mM, Labsynth, cat. no. C2010.01))
1.14 g Na_2_HPO_4_ (8 mM, Labsynth, cat. no. 01F2254.01)
0.59 g KH_2_PO_4_ (1.4 mM, Labsynth, cat. no. 01F2002.01)
1 L deionized water.
Adjust pH to 7.2‐7.4.
Store at room temperature

### Solutions A

4.2 g anhydrous citric acid (C_6_H_8_O_7_; Labsynth, cat. no. 01a1026.01)
200 ml sterile distilled water

### Solution B

52.92 g tribasic sodium citrate (Na_3_C_6_H_5_O_7_; Labsynth, cat. no. 01c1033.01)
1.8 L of sterile distilled water
Adjust the pH of solution B by adding drops of solution A (see recipe) until the pH is 6.0.
Store up to 1 week at 4°C.
  To adjust the pH to the desired value, typically 10% (v/v) of Solution A is added to the final volume.

## COMMENTARY

We developed this protocol due to the need for a reliable angiogenic evaluation tool in our laboratory. The various improvements made in this protocol have resulted in an easy and friendly procedure with impressive recovery rates, especially for the quail egg technique. To obtain good recovery rates, it is important to consider two main elements: (i) egg quality and (ii) cleaning steps. First, it is fundamental to have a trusted farm or company as a partner. Eggs should be from young quails and chickens under good treatment conditions to maximize egg quality. Regarding cleaning, contamination is one of the main reasons for egg loss in this experiment. Thereby, this procedure was designed to ensure the cleanliness and sterile state of the eggs throughout the entire process, starting with cleaning the eggs gently with 70% ethanol, using a sterile hood for all steps, and keeping the eggs in their shell.

Chicken embryos have a poorer survival rate than quail embryos during this type of experiment. We have had good recovery rates (above 40%) until the end of the procedure, but we believe that a partnership with a good producer will improve this even more. The use of quail eggs is poorly described in the literature, but our protocol was able to provide an outstanding survival rate for quail eggs (98%) from start to end. This reinforces the value of their application based on notable reproducibility, high survival rates, and faster handling due to their small size.

What makes this protocol different is the ability to scale up the experiment. In our routine work, we usually evaluate different biological materials that have been widely used in cancer, biotechnology, and bioengineering research for multiple research questions. We validated this protocol using up to 150 quail eggs and 50 chicken eggs, allowing reproducible results.

### Critical Parameters

#### Egg quality

As mentioned earlier, egg quality is the primary challenge with this technique, and collaborating with specialized producers is crucial for addressing this issue. However, there are additional important steps that can support the experiment. From day 1, when we obtain the eggs, the first crucial step is to store them in a room‐temperature environment (20°C‐23°C) while ensuring that they are kept away from dirt and sunlight. Avoid waiting too long to incubate the eggs; this will reduce the survival rate. During the cleaning procedure on the day of incubation, be cautious about using excessive ethanol, as it could be toxic to the embryo. Throughout the entire experiment, from the opening day to the stimulation day, avoid keeping eggs at temperatures other than their appropriate incubation temperature for very long by splitting them into small groups for the experiment.

#### Contamination problems

Because this is not a completely sterile procedure, it is important to be as cautious as possible. Starting from step 1, we described how important it is to keep everything clean and sterile during the procedure. Contamination episodes could happen at any time, even if everything is clean. If that occurs, save the healthy embryos and stop the experiment immediately to avoid spreading the problem. We recommend cleaning the incubator and hood using bleach (or 10% sodium hypochlorite solution) and 70% ethanol (after the bleach has dried properly) in sequence. Try to investigate the possible cause of the contamination, which could be one piece of dirty material used during the procedure or even the eggs from a specific batch. As described during the protocol, use all efforts to clean everything before starting the procedure. Remember to clean the bench where you leave the eggs until incubation day, clean the place where you keep eggs during incubation, sterilize everything that can be sterilized, and clean anything that cannot be sterilized properly with ethanol and UV light. As a last tip, when performing the procedure, dress in clean gloves, mask, cap, and lab coat.

### Troubleshooting

Troubleshooting advice can be found in Table 1. In general, it is important to highlight that the whole procedure is very delicate. Before performing an important experiment using quail and chicken chorioallantoic membrane, it is essential to spend time standardizing each step of this procedure while working with only a few embryos.

**Table 1:** Troubleshooting Description and Possible Solution

Step	Trouble	Potential solution
Egg opening procedure	Embryo viability is low	Incubate egg until 3‐5 days after collection to maintain viability at ∼80%. Make sure that there is no temperature variation during experimentation. Eggs must be stored at 36.5°C to 37.5°C.
Egg opening procedure	The membrane is glued to the shell	Use an incubator with rotation capacity to turn eggs at least once per day until the opening procedure is performed. If you do not have a rotating incubator, turn eggs sideways for at least 30 min before opening it up, and open it from the side instead of the top.
Histology processing	Tissue will not unstick from the filter paper envelope to be transferred into the paraffin	Use a heating platform; put the filter paper envelope on top of it and carefully use a spatula to lift off the membrane. Take care to not heat too much, which can cause loss of all paraffin in the tissue.
Tissue sectioning	Problems occur with sectioned tissue after paraffin embedding	Try hydrating the tissue paraffin block by placing it in a beaker of water and refrigerating it for ∼30 min. Try replacing the razor with a new one. If none of these solutions proves effective, the tissue probably was poorly processed at the initial steps, and it is advisable to process a new tissue. Another tip, although not part of standard histological protocols, is to add a small amount of beeswax or pure honey to the melted paraffin during the embedding step (1 hr in the second paraffin container), which may improve sectioning quality. While developing this protocol, we encountered this problem with one batch, and we subsequently tested the honey technique, confirming its potential effectiveness.
Histology staining	Slides have excess paraffin during histology staining	Slides may not have been in the incubator long enough to deparaffinize before staining. Try leaving a little more time for the xylene step.
Immunohistochemistry	Tissue section detaches from the slides during antigen recovery procedure in immunohistochemistry	Try readjusting the antigen recovery time according to the microwave's potency. Usually, the best solution is to shorten the microwaving time. Test silanized slides from a different brand as an alternative.
Scanning electron microscopy	Tissue melts during SEM analysis	The tissue probably was not dehydrated properly. Try increasing the alcohol wash time. If this does not work, the problem could be incomplete fixation. Try increasing the time in PFA after sample harvesting. If these suggestions do not work, try changing the fixative to Karnovsky solution (described in the Reagents and Solutions).

### Understanding Results

**Figure 7:**
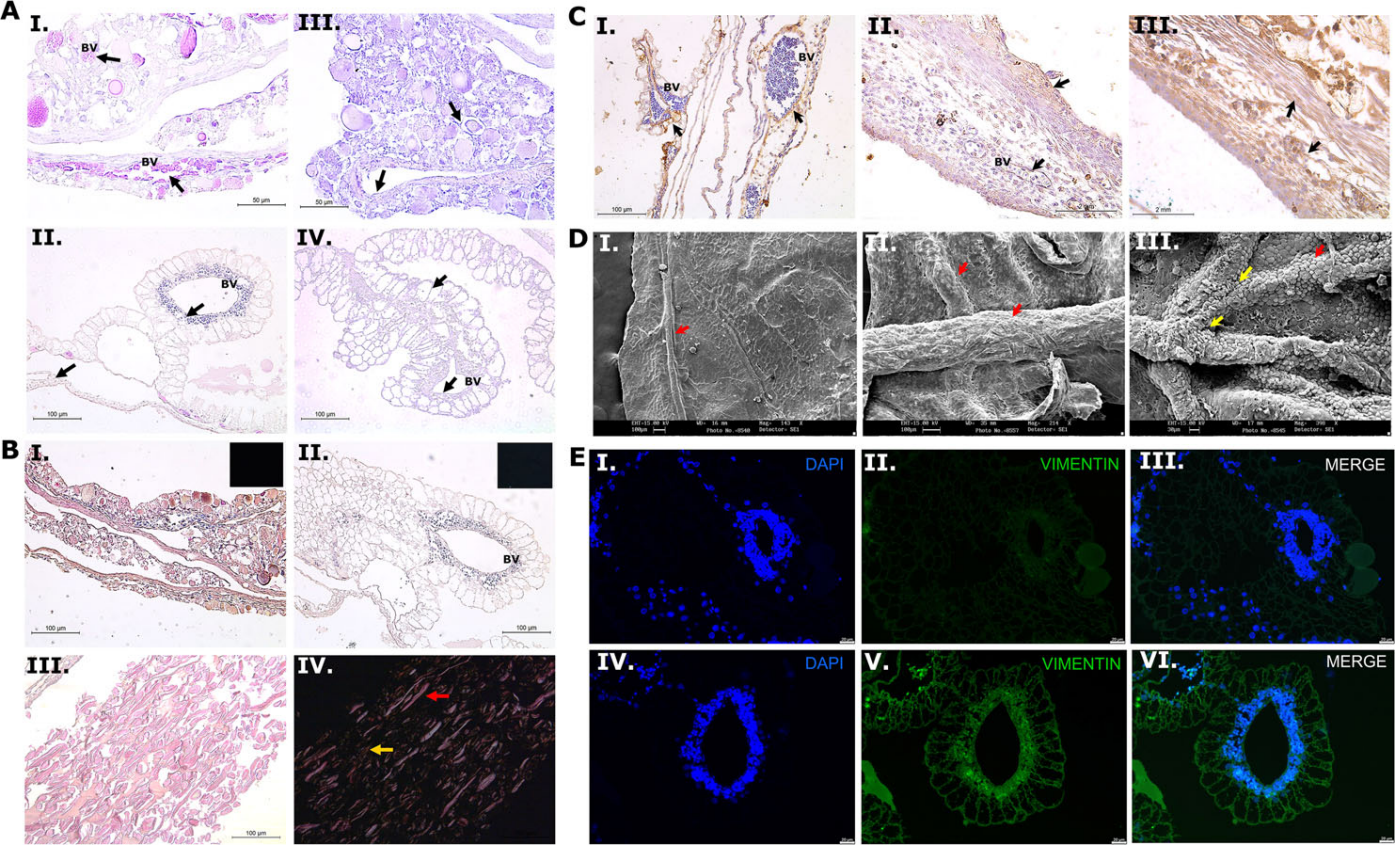
Representative images of the membrane 11 days into the experiment. (**A**) Hematoxylin and eosin (H&E) staining of chicken (I) and quail (II) membranes, showing the presence of blood vessels (BV) containing cells (indicated by arrow). Resorcin‐fuchsin staining demonstrates the presence of elastic fiber‐enriched regions in chicken (III) and quail (IV) membranes. (**B**) PicroSirius Red staining of membranes from chicken (I) and quail (II), showing lower collagen deposition in quail. In chicken, collagen deposition at this developmental stage was predominantly located in the allantoic epithelium (III and IV), with yellow‐red fibers (arrows) being indicative of thick, well‐organized collagen fibers involved in structural support. (**C**) Immunohistochemistry assays highlight specific structural proteins, including collagen I (I), collagen V (II), and MMP2 (III). (**D**) Scanning electron microscopy (SEM) analyses of vessel morphology show that on the external side of the membrane (I), specific vessel structures are poorly observed, whereas on the fetal side, sprouting areas (yellow arrow) and visible blood cells (red arrow) are clearly identified in both chicken (II) and quail (III). (**E**) Immunofluorescence staining to assess cellular presence using DAPI (I and IV) and vimentin (II and V); panels I, II, and III correspond to negative controls.

In our research, the CAM assay has been applied to investigate the angiogenic potential of conditioned medium and extracellular vesicles (International Society for Extracellular Vesicles, 2023). After membrane stimulation, vessel formation was observed using a stereomicroscope and then analyzed using hematoxylin and eosin staining to understand whether the extracellular vesicles promoted proliferation and vessel formation (Fig. 7A, panels I and II). In sequence, the elastic and collagen fibers were assessed by resorcin‐fuchsin (Fig. 7A, panels III and IV) and PicroSirius Red staining (Fig. 7B); elastic fibers could be seen mostly in the chicken membrane (Fig. 7A, panel III). However, collagen fiber staining suggested that quail embryos still do not have enough deposition of these fibers in most regions to be evaluated in a nonspecific analysis (Fig. 7B, panel II). Interestingly, it was possible to see that PicroSirius Red revealed strong collagen deposition in chicken membranes at this development stage in the allantoic epithelium (Fig. 7B, panel III), with polarization of yellow‐red fibers Fig. 7B, panel IV). We also noted that the evaluation of collagen through immunohistochemistry using specific antibodies is much more accurate than its evaluation using histology. In the same way, IHC is a good alternative method to investigate pro‐ and anti‐angiogenic potential, not only to visualize vessels using CD31 and VEGF antibodies but also to investigate structural proteins involved in angiogenesis signaling pathways, such as metalloproteinases, elastin, different types of collagens, and laminin (Fig. 7C). In addition, we demonstrated using SEM that the CAM membrane presents differences between the maternal‐fetal and external zones (Fig. 7D): blood vessels can be seen specifically on the maternal‐fetal side (Fig. 7D, panels II and III), whereas the external side (Fig. 7D, panel I) shows some wrinkles but is mostly flat. Cell presence and structure were also evaluated using immunofluorescence (Fig. 7E). Although this is not described in this protocol, immunostaining data can be quantified using ImageJ software with a simple thresholding setting.

### Time Considerations

In general, this protocol takes ∼20 days, including preparation and membrane analyses. However, as described above, the procedure is separated into sections, and each of them takes a different time. Calculations were made considering an average of 30 eggs.

**Table:** 

Experiment section	Time
Cleaning and setup of the incubator	30 min
Cleaning and incubation of eggs	30‐40 min
Egg opening	1‐2 hr
Treatment and collection of chorioallantoic membrane	30 min
Preparation of chorioallantoic membrane for histological processing	5‐6 hr
Histological processing	3‐4 hr
Histological staining	2 hr per stain
Imaging analysis	SEM, 1 hr; IHC/IF, 2 days; imaging time depends on the number of samples

### Author Contributions

**Letícia Alves Fernandes**: Formal analysis; investigation; methodology; visualization; writing—original draft. **Gabriela Riceti Inhauser Magalhães**: Data curation; investigation. **Ana Claudia Oliveira Carreira**: Conceptualization; funding acquisition; resources; supervision; validation; writing—review and editing.

### Conflict of Interest

The authors declare no conflict of interest.

## Data Availability

All data obtained from untreated samples are available and have been shown in this manuscript.
